# Efficient CORDIC Iteration Design of LiDAR Sensors’ Point-Cloud Map Reconstruction Technology †

**DOI:** 10.3390/s19245412

**Published:** 2019-12-09

**Authors:** Yu-Cheng Fan, Yi-Cheng Liu, Chiao-An Chu

**Affiliations:** 1 Department of Electronic Engineering, National Taipei University of Technology, Taipei 10608, Taiwan; a0910102082@gmail.com; 2 Sunplus Technology Co., Ltd., Hsinchu 30076, Taiwan; alice.chu@sunplus.com

**Keywords:** autonomous car, CORDIC, LiDAR, point cloud

## Abstract

In this paper, we propose an efficient COordinate Rotation DIgital Computer (CORDIC) iteration circuit design for Light Detection and Ranging (LiDAR) sensors. A novel CORDIC architecture that achieves the goal of pre-selecting angles and reduces the number of iterations is presented for LiDAR sensors. The value of the trigonometric functions can be found in seven rotations regardless of the number of input N digits. The number of iterations are reduced by more than half. The experimental results show the similarity value to be all 1 and prove that the LiDAR decoded packet results are exactly the same as the ground truth. The total chip area is 1.93 mm × 1.93 mm and the core area is 1.32 mm × 1.32 mm, separately. The number of logic gates is 129,688. The designed chip only takes 0.012 ms and 0.912 ms to decode a packet and a 3D frame of LiDAR sensors, respectively. The throughput of the chip is 8.2105${\;\times \;10}^{8}$ bits/sec. The average power consumption is 237.34 mW at a maximum operating frequency of 100 MHz. This design can not only reduce the number of iterations and the computing time but also reduce the chip area. This paper provides an efficient CORDIC iteration design and solution for LiDAR sensors to reconstruct the point-cloud map for autonomous vehicles.

## 1. Introduction

Technology is constantly changing and evolving to make human life more convenient and comfortable, and automation will be a major trend in future evolutions of technology. In the area of automation, a major development trend in the next few years will be autonomous cars [1].

Because autonomous driving is the future trend and Light Detection and Ranging (LiDAR) is the core of autonomous driving, one of the key themes in autonomous car research in LiDAR [2,3,4,5]. Dimitrievski considers that LiDAR is 360 degrees surround sensor while the camera is a single view frontal view sensor [2]. Therefore, a LiDAR sensor can detect objects that are located on the sides and back of the ego vehicle [2]. Dimitrievski uses the results of 3D LiDAR measurements in the data association function, resulting in a notable increase in robustness to person-to-person and person-to-background occlusions [2]. Moreover, Zhang adopts LiDAR sensors to perform “vehicle detection using probability hypothesis density filter [3].” Zhang considers that LiDAR is robust against light intensities and widely used in vehicle detection [3]. LiDAR provides a large number of measurements and tracks the potential objects without any association information or cluster process [3]. In addition, Shahian Jahromi explains that LiDAR measures the surrounding environment containing the position (x, y, z coordinates) and intensity information of the objects [4]. LiDAR adds angular resolution (horizontal azimuth and vertical) for better measurement accuracy compared to the camera and radar sensors by measuring range [4]. Therefore, LiDAR is a core sensor that is used for autonomous vehicles [4]. Furthermore, LiDAR is a very powerful sensor with wide scanning range that can capture the depth value, distance and contour information of objects precisely [5]. At the same time, LiDAR is not easily affected by environmental interference and provides fast and accurate measured results [5]. For these reasons, many developers use this for autonomous vehicles. LiDAR-scanned packet information can provide distance, angle, and reflectivity information, and such information can be reassembled to construct a point-cloud map [2,3,4,5]. In decoding the LiDAR sensor packet, the 3D coordinates must be calculated and the COordinate Rotation DIgital Computer (CORDIC) algorithm must be used to solve the sine (SIN) and cosine (COS) values. However, the traditional CORDIC algorithm requires a large number of iterations and entails a long delay time. If accuracy of N bits is required, N iterations must be performed. Such an algorithm is not suitable for autonomous-driving circuits, which require high-speed processing. However, if we use the look-up table (LUT) scheme [6], the circuit area will become larger. Therefore, the number of CORDIC iterations and the area of the CORDIC circuit need to be reduced for LiDAR sensors. To solve the above problems, this paper proposes an efficient CORDIC iteration circuit design for LiDAR sensors to reconstruct a point-cloud map application to autonomous driving.

This paper is composed as follows. Section 2 describes the related work on LiDAR sensors and CORDIC circuits. Section 3 presents the LiDAR sensing system, LiDAR packet decoding, 3D point-cloud map reconstruction and the CORDIC iterative reduction algorithm. Then the mobile LiDAR sensing circuit and chip design are addressed in Section 4. In Section 5, experimental results are presented, and conclusions are stated in Section 6.

## 2. Related Work

Three-dimensional LiDAR [7,8,9,10,11,12,13,14,15,16,17] sensors mainly use pulsed laser light emitted by a laser emitter to detect the environment or measure an object. The working principle is that laser light is emitted by LiDAR and hits the object. The resulting scattering and reflection phenomena are due to the reflection of light, and LiDAR sensors receive the signal [9]. The distance information is obtained from the known speed of light. Through the reflection phenomenon of light, the smoothness and color of the surface of the object hit by the beam can be known. In general, objects have a reflectivity of 0 and 1 for light, but the reflectivity can be subdivided into 256 levels (0–255) in a LiDAR packet. In LiDAR, the reflectance can be divided into diffuse reflection and total reflection.

Diffuse reflection is defined as the phenomenon wherein light is reflected out of order when light hits the surface of a rough object. When the light hits a rough black surface, the reflectance is 0 because a black surface absorbs light. When the light hits a rough white surface, the reflectance is 1 to 100.

Total reflection is defined as the light being reflected directly back when the light hits a smooth surface, such as a mirror. When the light hits a reflector without any coverage, the reflectivity is 255. When the light hits a translucent covered reflector, the reflectivity is 101 to 254.

In this paper, the data receiving method of the LiDAR sensor is divided into three modes. The first is the Strongest Return Mode, which is the information returned when the beam hits a near object, which is equivalent to receiving the strongest beam of infrared light. The second type is Last Return Mode; when the beam hits a far object, the information is returned. The third type is the Dual Return Mode, which means that the information includes both last return and strongest return information.

After LiDAR detects the environmental information, a circuit needs to decode the collected LiDAR packet data and then decode the packet into 3D coordinates using the CORDIC algorithm [18]. The relevant literature on the CORDIC algorithm and circuit design and the explain the problems that exist in the current relevant research are described below.

Traditional CORDIC algorithms use basic addition, subtraction and shifting to apply trigonometric functions and other basic arithmetic, such as multiplication and division [1].

The CORDIC algorithm was first proposed by J.E. Volder in 1959 mainly for trigonometric functions [19]. In 1971, J.S. Walther applied the algorithm to hyperbolic and exponential functions, to multiplication and logarithms, and other functions. In 1974, A.M. Despain used the CORDIC iterative principle to perform the Fourier transform [20].

The principle of a CORDIC algorithm [21] is to use the rotation of a two-dimensional coordinate plane to complete many complicated operations. The hybrid adaptive CORDIC algorithm (HA CORDIC) [21,22,23,24] is mainly used to reduce the number of iterations of traditional CORDIC rotation [1]. We assume that any point of the two-dimensional plane coordinates is the origin (X_0_, Y_0_) rotated to (X, Y), and the angle of rotation is θ. We can write the rotated matrix. We decompose the angle of rotation θ into a number of small associated angles and set these small angles as α angles. If the sum of all the α angles is equal to the original rotation angle θ, then in the rotated matrix the θ angle can be replaced with the α angle. [1]

When the coordinate rotation angle is equal to some specific rotation angle, then to realize the simple displacement operation in the digital circuit design, let tan$\alpha_{i}=\sigma_{i}2^{-i}$ and K = $\prod_{i=0}^{n-1}\cos\alpha_{i}$. After the substitution, Formula (1) can be obtained.

(1) $$\left[\begin{array}{c} X^{\prime }\\ Y^{\prime } \end{array}\right]=K\left[\begin{array}{cc} 1 & \sigma_{i}2^{-i}\\ \sigma_{i}2^{-i} & 1 \end{array}\right]\left[\begin{array}{c} X_{0}\\ Y_{0} \end{array}\right]$$

(2) $$Z_{n}=\theta \sum_{i=0}^{n-1}\sigma_{i}\tan^{-1}2^{-i}$$

Finally, the matrix of Equation (1) is expanded, and from Equation (2), then Equations (3)–(5) can be obtained.

(3) $$Z_{i+1}{=Z}_{i}-\sigma_{i}\tan^{-1}2^{-i}$$

(4) $$X_{i+1}{=X}_{i}-\sigma_{i}{\;Y}_{i}{\;2}^{-i}$$

(5) $$Y_{i+1}{=Y}_{i}-\sigma_{i}{\;X}_{i}{\;2}^{-i}$$

In the above formula, σ is the direction of the selected rotation angle, clockwise rotation is a positive sign, and counterclockwise rotation is a negative sign. Z is the sum of the angular rotation operations. The final calculation results need to make the angle of the sum after Z rotation approach zero.

The number of iterations is determined by the number of bits. For example, if the input angle Z is 16 bits, it needs to be rotated 16 times. X represents the result of the COS operation and Y represents the result of the SIN operation. Assuming the input angle is 30 degrees, Z will approach zero after the rotation. X will approach 0.866 and Y will approach 0.5.

After a series of rotations, the rotated vector will be different from the original vector. To make the rotated vector equal to the original vector, it is necessary to multiply the vector factor K of the corrected length.

Let tan$\alpha_{i}{=\;\sigma }_{i}2^{-i}$ and K = $\prod_{i=0}^{n-1}{\cos\alpha }_{i}$. With the Pythagorean theorem, we can calculate $\cos=\;\frac{1}{\sqrt{{1+2}^{-2i}}}$ and bring it into the K related formula. Finally, after expanding the infinite series of K, the approximate constant value is 1.64676, as shown in Equation (6).

(6) $$K\;=\prod_{i=0}^{n-1}\left(\frac{1}{\sqrt{{1+2}^{-2i}}}\right)=1.64676$$

For the calculation of CORDIC, many iterations are required. In the literature, many papers propose ways to reduce the number of iterations.

HA CORDIC [22,23,24] is mainly used to reduce the number of iterations of a traditional coordinate rotation digital computer. The principle is to use the traditional coordinate rotation system to fix the angle between the two angles. The angle θ is a fixed angle, and c(0) is 45 degrees plus 26.565051177 degrees. After addition, the value is divided by 2 to 35.782525588 degrees, and the values of c(0) to c(15) are obtained in this way. Z(i) represents the input rotation angle, and N represents the value of inputting a few bits, indicating that the maximum number of rotations is only N. The two variables are set to i and j, respectively, and the initial value is set to zero. When the first input angle Z(0) is greater than c(15) and j is less than N, the condition is established and the loop is established. Finally, i will be equal to i plus 1. If the condition is not established, it will not jump to the loop. j will be equal to j plus 1. By changing the two variables i and j into Formulas (7)–(9), the pre-selected iteration angle can be achieved.

(7) $$Z_{i+1}{=Z}_{i}-d_{i}\theta_{j}$$

(8) $$X_{i+1}{=X}_{i}-d_{i}{\;Y}_{i}{\;2}^{-j}$$

(9) $$Y_{i+1}{=Y}_{i}-d_{i}{\;X}_{i}{\;2}^{-j}$$

The HA CORDIC algorithm uses the angle between the two intermediate values of the fixed angles to compare the comparison values, and the input angle is compared to the intermediate values, thus achieving the function of pre-rotation angle.

In [25], Qi and Cabe proposed a CORDIC processor with three computation modes. The CORDIC core uses 16-bit fixed point numbers. Qi and Cabe performed one iteration per bit of the input data. In this circuit, 16 vector rotations are needed and 16 stages are constructed in the CORDIC pipeline circuit. Wu and Shiue [26] proposed a field programmable gate array (FPGA) prototype of a CORDIC operation. The circular and linear rotation of the unified CORDIC are proposed to fulfill the derotator [26]. Ray and Dhar presented a CORDIC-based unified Very Large Scale Integration (VLSI) architecture that uses a parallel pipeline architecture with latency equal to twice the CORDIC length plus three extra cycles [27]. It includes a linear CORDIC and circular CORDIC with first in and first out register (FIFO) [27].

Meng and Wang proposed a rotary encoder using the CORDIC algorithm to calculate the arctangent with an iterative technique to compute hyperbolic and trigonometric functions. The modified CORDIC algorithm reduces the iteration time. However, the changes of the error margin are similar [28].

Xia and Yu designed a CORDIC algorithm to compute trigonometric functions [29]. This method conserves resources and reduces the power dissipation in FPGA [29]. The design makes full use of the CORDIC algorithm in rotation mode to obtain the sin θ and cos θ values [29]. A 16-level pipeline structure CORDIC is realized in FPGA, which uses 16 layers of modules [29]. The inner CORDIC module is serially cascaded [29].

However, the traditional CORDIC algorithm is tedious and time consuming. The area will become larger if a look-up table is used. The schemes in the literature are not suitable for small-sized or fast circuit designs.

## 3. Proposed Method

The mobile LiDAR sensor circuit design is mainly used in autonomous vehicles and environmental sensing. Due to the trend of autonomous vehicles development in the future, the main goal of this paper is to design a high-performance mobile LiDAR sensing circuit to realize LiDAR packet decoding and improve the traditional CORDIC architecture to simplify the operation of 3D LiDAR point-cloud map reconstruction. In this section, we propose an algorithm that simplifies the number of CORDIC rotation iterations. We explain in turn how the packet is decoded and how the features of CORDIC are employed to reduce the iterative operation.

### 3.1. Light Detection and Ranging (LiDAR) Sensing System Overview

We use LiDAR to scan environmental information in a 3D space. After the LiDAR scan, the packet is taken out, and the information such as distance, angle and reflectivity can be obtained after the packet is decoded. We use the packet information to construct a three-dimensional point-cloud map.

### 3.2. Three-Dimensional (3D) Mobile LiDAR Packet Decoding

In the process of converting the packet information into a three-dimensional point-cloud image and improving the rotation angle of the algorithm, we first need to decode the packet information scanned by LiDAR. From the decoding step, we obtain the angle, distance, reflectivity and other information of the three-dimensional space in the environment. According to the angel information, we calculate the values of SIN and COS through the proposed CORDIC iterative reduction algorithm. The X, Y, and Z values are calculated by using the three-dimensional space construction formula and then converted to construct a three-dimensional point-cloud map.

In the case of a packet, the first data block will be Last Return data, and the second data block will be Strongest Return data. Selecting the Dual Return Mode will return two reflectance information at the same horizontal angle for every two blocks. Therefore, for the same horizontal angle, different data-receiving modes are selected, and the amounts of data received will be different. Selecting Dual Return Mode will double the amount of information returned by the Strongest Return and Last Return modes.

Table 1 shows the packet rate for a single packet data received by the Strongest Return Mode or Last Return Mode. When the single-return method is selected, the data volume receives 754 packets and 8 Megabits per second. If we the select Strongest Return and Last Return Modes at the same time, twice the amount of data will be received.

**Figure 1:**
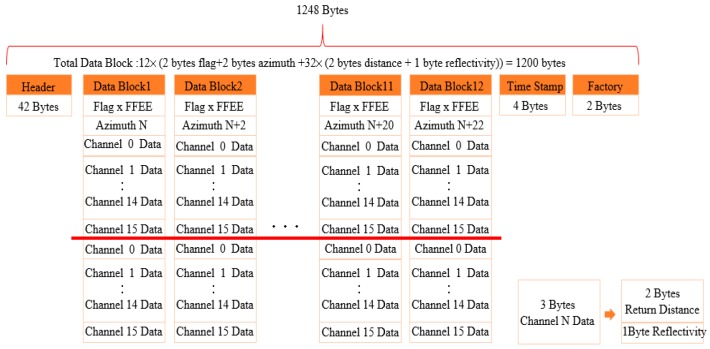
Mobile LiDAR data packet.

LiDAR’s data packet uses the user datagram protocol (UDP) to transmit data. A packet will have a total of 1248 bytes and contain 42 bytes of header files. The 12 Data Blocks have a total of 1200 bytes and record information such as angle, distance, and reflectivity. An additional 4 bytes of timestamp record Global Positioning System (GPS) information. Finally, the 2 bytes of factory data are used to record which mode is used and the LiDAR model. A Data Block can be divided into 2 bytes of Flag, 2 bytes of horizontal angle information, and two sets of 0 to 15 channel data. One channel data is 3 bytes, which contains the information of the first 2 bytes of distance, and the reflectivity information of the last 1 byte, as shown in Figure 1.

The LiDAR horizontal angle information is contained in the Data Block, and each Data Block contains two different horizontal angles. The first set of horizontal angles can be derived from the packet information before the first Channel 0 Data. The second set of horizontal angles, also known as drift angles, is taken before the second Channel 0 Data. To calculate the second set of drift angles, we use the first Data Block plus the horizontal angle of the second Data Block and divide by two, as shown in Equation (10).

(10) $$\mathrm{Angular}\;\mathrm{Drift}\;=\;\frac{\mathrm{Data}\;\mathrm{Block}\;0\;+\;\mathrm{Data}\;\mathrm{Block}\;1}{2}$$

**Figure 2:**
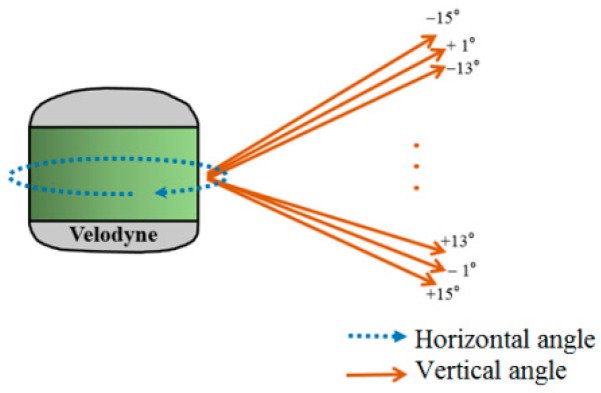
Mobile LiDAR horizontal and vertical angle.

The vertical angle of the LiDAR is fixed. When the light is rotated, it will continuously rotate the horizontal angle by 360 degrees at 10 times per second and emit 16 rays at the same time, as shown in Figure 2. The vertical angle corresponding mode starts from Channel 0 and goes down to Channel 15, corresponding to the LiDAR vertical angles in Table 2. The laser beam from the zeroth pass is −15°, the first laser beam is +1°, the fifteenth laser beam is +15°, and each vertical laser beam is separated by 2 degrees.

**Figure 3:**
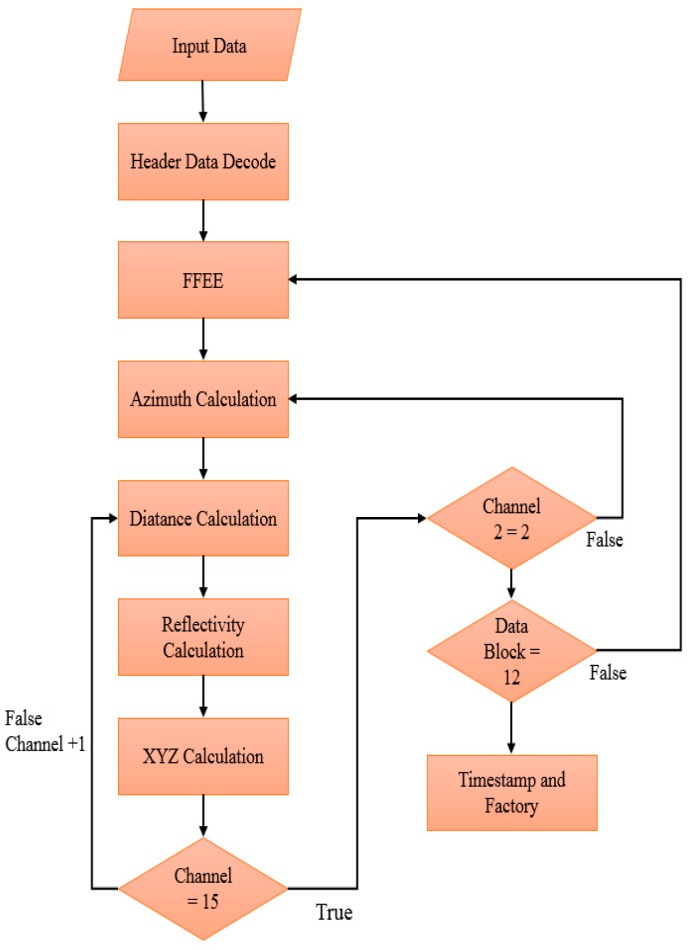
LiDAR decoding flow chart.

The decoding process is shown in Figure 3. The LiDAR sensor circuit sequentially solves the information such as the header, flag, horizontal angle, distance, and reflectivity. The data input is a continuous 2 bytes of information. After the correctness of the header at the beginning of the packet is checked, the packet decoding will begin. First, the circuit confirms that each block’s flag is FFEE information of 4 bytes in succession. After confirmation that the information is correct, the horizontal angles of 4 consecutive bytes are calculated, followed by calculation of the distance information, reflectivity information, and 3D point XYZ, and then confirmation of whether a Data Block has two sets of 0 to 15 channels. The process will not continue to calculate the Channel information of the current Data Block. After a complete calculation of a set of Data Blocks, the process will confirm whether 12 sets of Data Blocks have been calculated and finally check the GPS and LiDAR model information.

### 3.3. Three-Dimensional Point-Cloud Map Reconstruction

The 3D point-cloud map consists of a number of points of the 3D vector coordinate Data Point. Therefore, we can obtain the R-distance and the α-horizontal angle after decoding the LiDAR packet, and find the corresponding Channel Data to know directly that ω is the vertical angle. With this information, the XYZ coordinates are calculated and the location of the Data Point is known, and the point-cloud map information is reconstructed according to the Data Point of each point.

**Figure 4:**
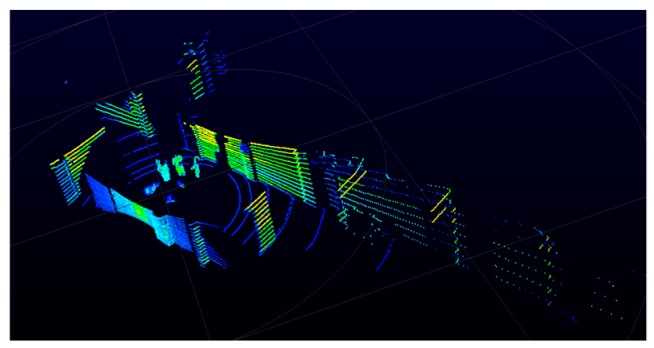
Three-dimensional point-cloud map.

LiDAR is an adjustable design with 5–20 turns per second. This paper uses 10 rotations per second and obtaining a complete frame requires 75 packets of information, each having a horizontal angle of 4.8 degrees. After all the packet information is decoded, a complete picture can be obtained. Figure 4 shows the 3D point-cloud image information obtained after 75 packets are decoded.

### 3.4. COordinate Rotation DIgital Computer (CORDIC) Iterative Reduction Algorithm

The traditional CORDIC algorithm is tedious and time consuming. However, if the look-up table method is used, the area will become larger. A larger area is less suitable for circuits that require a small size or high speed. In this paper, a new method of angle selection is proposed. We use the rotation input angle in the formula to rotate to the end, which must be close to zero to pre-select the desired iteration angle to reduce the number of iterations.

**Figure 5:**
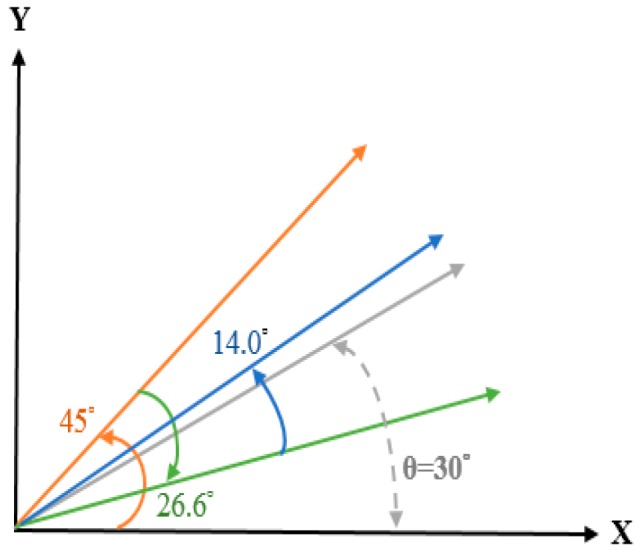
The rotation direction of the traditional COordinate Rotation DIgital Computer (CORDIC) algorithm.

From the last formula derived from the traditional algorithm, it can be known that X and Y represent the values of COS and SIN, respectively. The angle to be searched for is the input angle Z, and each rotation will use a fixed angle of θ. For example, the first input angle will increase or decrease the angle by 45 degrees. The second time will increase or decrease the angle by 26 degrees. To select the desired angle, we use the angle Z that we are looking for, and the result of the final sum needs to approach zero, as shown in Figure 5. Table 3 shows a list of traditional CORDIC algorithm rules. The traditional CORDIC algorithm has a number of rotations ***i***, and the degree of addition and subtraction angles is θ. X and Y are closer to the values of the angle sought after each operation. That is to say, the rotation angle selection of Z is the key to the number of iterations. Therefore, the number of iterations ***i*** can be changed by changing the addition and subtraction angle calculation of the angle Z at the beginning.

It can be known from the traditional algorithm formula that the number of iterations can be changed by changing the angle selection mode of Z. This paper uses this feature to compare the input angle Z to the highest bit of the fixed input θ angle. When the highest bit of the Z angle is greater than or equal to the highest bit, the Z angle will subtract the θ angle. If this condition is not true, it will continue to compare the highest bit of the θ angle of the next fixed input. It will first compare the maximum specific angle of the input and the highest bit of 4, and then compare the highest level of the input angle of the next order, and compare them sequentially (Table 3). When the angle is input and compared, the input angle and the fixed angle are added and subtracted. If the remaining residual value is less than the minimum angle of the fixed θ angle after the calculation, the loop is jumped out. If the final residual value is 0.001, then the loop is jumped out of. If the condition is not met, the angle comparison will be restarted until the residual value is less than 0.001.

**Figure 6:**
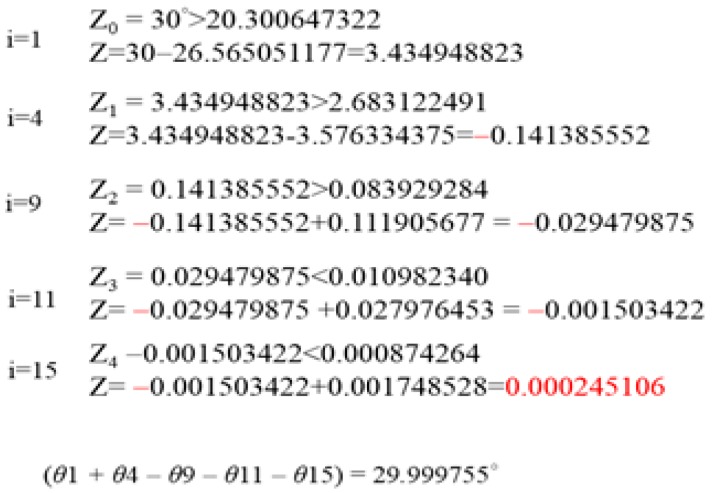
Example of angle selection.

Taking the actual input angle as an example, suppose that there is an input angle Z of 30 degrees, and under this condition, the result of Z must approach zero. According to the flow chart, first, the highest bit value of the input angle is 3, and the highest bit value of the fixed θ angle is 4. Because the highest bit value 3 of the input angle is less than the highest bit value of the fixed comparison angle 4, the condition is not true. Therefore, the highest value of the input angle will be compared to the highest value of the next input fixed θ angle value 2. After the angle comparison condition is true, the input angle of 30 degrees will be reduced by the fixed angle of 26.565051177 degrees, and the remainder is 3.343948823 degrees. At this time, the input angle value 3 will be re-compared with the fixed angles of 45 degrees, 26 degrees, and 14 degrees. Since these values are all tens of decimals, the condition will not be true. Then we continue to compare the value 7, because the residual value 3 of the input angle is smaller than the value 7 of the fixed comparison angle, the condition is not true. Then we continue to compare the fixed angle value 3; the input angle residual value 3 is equal to the fixed input angle 3, so the angle will do the subtraction action. After the subtraction, the residual value is negative 0.1, so we continue to find a fixed input angle equal to or less than 0.1. Finding a fixed input angle of 0.111905677 will add up. The main principle of the traditional rotation angle is that the input angle must approach zero. Therefore, the last residual value is a negative value, the next search will be added, and the input angle will gradually approach the condition. When the final calculation is performed, whether the positive or negative residual value is not less than 0.001, the angle will continue to be compared until the residual value is less than 0.001 to jump out of the loop, as shown in Figure 6. In this way, the input angle Z is 30 degrees, and only five iterations are needed to find the required value.

The rotated vector will be different from the original vector. To make the vector length the same, the vector needs to be multiplied by the vector length correction factor. However, if the pre-angle selection method is used, the original value needs to be set to X = 1, Y = 0, so the vector correction factor must wait for the X and Y rotations before the K total product is multiplied after each iteration. In the traditional CORDIC algorithm, it can be known that for some specific angles, a simple shift instruction can be used, so let tan$\alpha_{i}=2^{-i}$ and K = $\prod_{i=0}^{n-1}\cos\alpha_{i}$. Through the Pythagorean theorem in the trigonometric function, we get $\;\cos\alpha_{i}=\frac{1}{\sqrt{1+2^{-2i}}}$. The total product of K has an iteration coefficient i for each iteration. Substituting i into Equation (11), we can obtain the vector length correction factor. Finally, the total product of K can be calculated as the sum of the number of iterations corrected each time. Taking the input angle of 30 degrees as an example, the selection angle selects the iteration angles of i = 1, 4, 9, 11, and 15, respectively, so the correction length factor K is K1 × K4 × K9 × K11 × K15. The K value of each iteration is shown in Table 4.

(11) $$K\;=\prod_{i=0}^{n-1}\left(\frac{1}{\sqrt{{1+2}^{-2i}}}\right)$$

## 4. Mobile LiDAR Sensing Circuit and Chip Design

In this section, we present the design of the LiDAR sensing circuit and chip. We will describe in detail the function of each block, including LiDAR packet decoding, the CORDIC calculation architecture, and the chip design. At the same time, the algorithm for reducing the CORDIC iteration is used in the CORDIC block to calculate the values of COS and SIN, and finally the XYZ coordinate value of the 3D point-cloud image is calculated.

### 4.1. System Architecture

**Figure 7:**
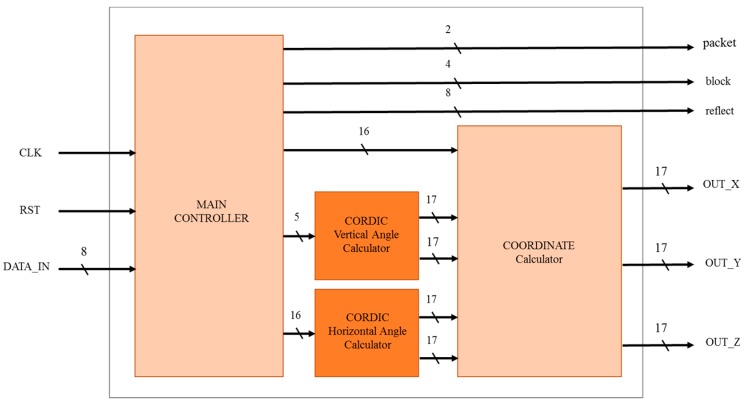
System architecture.

The mobile LiDAR sensing architecture can be divided into four parts, namely the Main Controller, CORDIC Vertical Angle Calculator, CORDIC Horizontal Angle Calculator, and Coordinate Conversion Calculation (Figure 7).

The main controller is primarily used to classify and decode input data. The Main Controller receives 8-bit LiDAR input signals, and outputs 2-bit packets, 4-bit blocks, 8-bit reflection information, and 16-bit distance information to the coordinate conversion calculator. The vertical angle and horizontal angle calculators are trigonometric operations.

The CORDIC algorithm is used to approximate the values to be obtained, the horizontal and vertical angles of SIN and COS are calculated, and the final output 17 bits is transmitted to the coordinate conversion calculator.

The final module coordinate conversion calculator is to receive the 16-bit distance information, the 17-bit SIN and COS values of the vertical angle, and the 17-bit SIN and COS values of the horizontal angle. The final XYZ coordinates are obtained by the formula operation.

### 4.2. Main Controller

The Main Controller begins counting the data when it receives the LiDAR data. First, the header file starts counting from 0 to 41. When the count reaches 41, the flag Flag 1 state is started. When cnt_b starts counting 1, it will notify Flag 1 to move to the state of Flag 2. When the cnt_b count is 2, Flag 2 will be notified to move to the horizontal angle 1 (Az1). When the horizontal angle receives a cnt_b count of 3, it will move to the horizontal angle 2 (Az2). The horizontal angle 2 will start to calculate the horizontal angle information. The horizontal angle output is an 8-bit Az1 signal combined with an 8-bit Az2 signal equal to a 16-bit horizontal angle, as shown in Equation (12).

Azimuth = {Az1,Az2}(12)

When the horizontal angle 2 (Az2) is calculated and the next step of cnt_b is received, it is moved to the distance 1 (Dist1) for calculation. When cnt_b changes again, cnt_b moves to distance 2 (Dist2) for calculation. After the calculation, the 8-bit distance 1 (Dist1) information is combined with the 8-bit distance 2 (Dist2) information and multiplied by 2 to be the 16-bit distance information, as shown in Equation (13).

Distance ={Dist1,Dist2} × 2(13)

**Figure 8:**
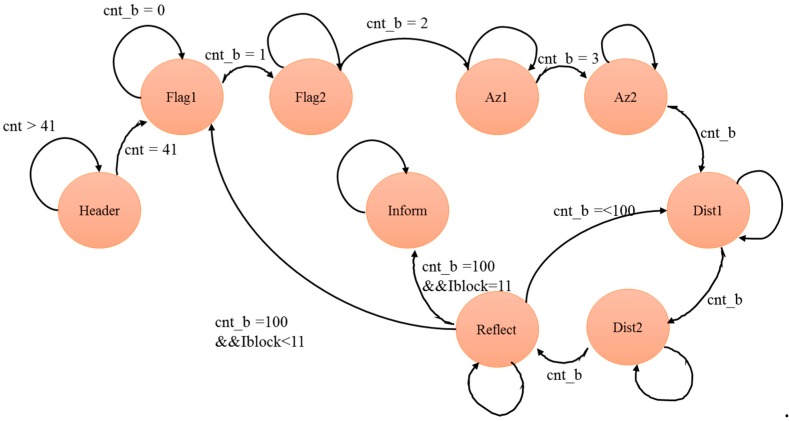
Finite state machine of main controller.

When the operation of distance information is completed, the cnt_b changes again to the Reflect state for calculation. If the cnt_b count value is less than 100, the main controller returns to the distance 1 (Dist1) state. If the cnt_b count value is 100 and the Iblock value is less than 11, then it returns to the flag 1 (Flag1). If the cnt_b count value is 100 and the Iblock value is equal to 11, then it returns to the LiDAR receiving information state until all information is received. Therefore, the main controller needs about 1716 cycles to decode one packet, as shown in Figure 8.

### 4.3. CORDIC Angle Calculator

The CORDIC angle calculator is the most occupied part of the hardware, and it also consumes the most power. According to the architecture designed in this paper, it can be divided into a CORDIC horizontal angle calculator and a CORDIC vertical angle calculator. The vertical angle input is a fixed input angle and the horizontal angle input is 360 degrees.

#### 4.3.1. CORDIC Horizontal Angle Calculator

LiDAR is a circular scanner with a horizontal angle of 360 degrees, so the horizontal input angle will be 0 to 360 degrees. There will be two horizontal angles in a block. The first horizontal angle can be determined from the packet information. The second horizontal angle is the horizontal drift angle. The entire packet has a total horizontal angle scan range of 4.8 degrees, and a packet has a total of 12 blocks, so each block has a horizontal angle of 0.4 degrees and a horizontal drift angle of 0.2 degrees.

**Figure 9:**
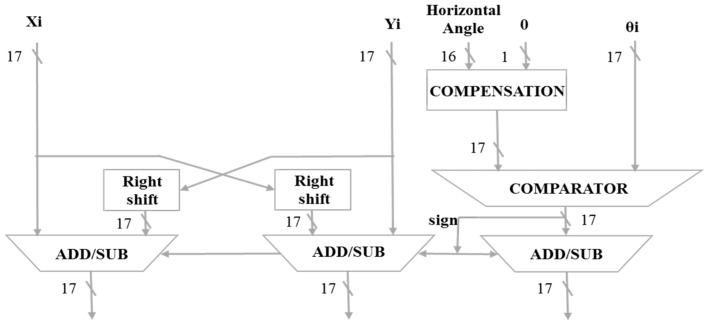
CORDIC horizontal angle calculator.

The CORDIC horizontal angle calculator is shown in Figure 9. When the 16-bit horizontal angle information is received, it will be compensated first for 17 bits. The ***Y_i_*** input information is 0. The ***X_i_*** input information is 1. ***θ_i_*** is a fixed comparison angle compared to the horizontal angle of the input. After the information is compared, the signal will be transmitted to shift and subtract the ***X_i_*** and ***Y_i_*** signals. The most significant bit (MSB) is used to determine whether it is a 2′s complement.

#### 4.3.2. CORDIC Vertical Angle Calculator

The vertical angle input is from positive 15 degrees to minus 15 degrees. The corresponding channel from LiDAR determines the degrees of vertical angle. However, since the angle is 15 degrees, the input will not be compared to the fixed angles of 45 and 26 degrees. Therefore, the angle selection can be directly reduced by 45 degrees and 26 degrees, and the relative iteration is also relatively fast.

**Figure 10:**
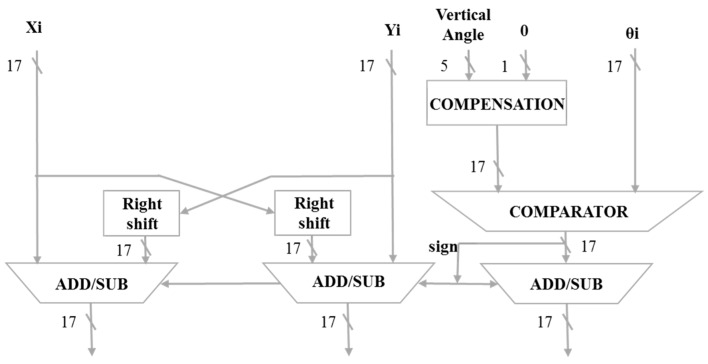
CORDIC vertical angle calculator.

The CORDIC vertical angle calculator is shown in Figure 10. When receiving the 5-bit angle information of the vertical angle, it will be compensated first for 17 bits. The ***Yi*** input information is 0. The ***Xi*** input information is 1. ***θi*** is a fixed comparison angle that will be compared with the vertical angle of the input. After the information is compared, the signal will be transmitted to shift and subtract the ***Xi*** and ***Yi*** signals. The highest bit MSB is used to determine whether it is a 2′s complement.

#### 4.3.3. Angle Normalization

Using characteristics of SIN and COS in four quadrants, all input angles are first standardized from 0 to 90 degrees. When the input angle is 0 to 90 degrees, the output is positive COS and positive SIN. When the input angle is 90 to 180 degrees, the input angle is subtracted by 180 degrees, and the output is negative COS and positive SIN. When the input angle is 180 to 270 degrees, the input angle is used to subtract 180 degrees, and the output is negative COS and negative SIN. When the input angle is 270 to 360 degrees, the input angle is subtracted by 360 degrees, and the output is positive COS and negative SIN. To normalize the angle to 0 to 90 degrees, the final result of the outputted COS and SIN must be plus or minus. Therefore, the output angle information is originally 16 bits, and one bit is added to the front of the 16-bit number. The output information is a total of 17 bits.

#### 4.3.4. XYZ Iteration Architecture

**Figure 11:**
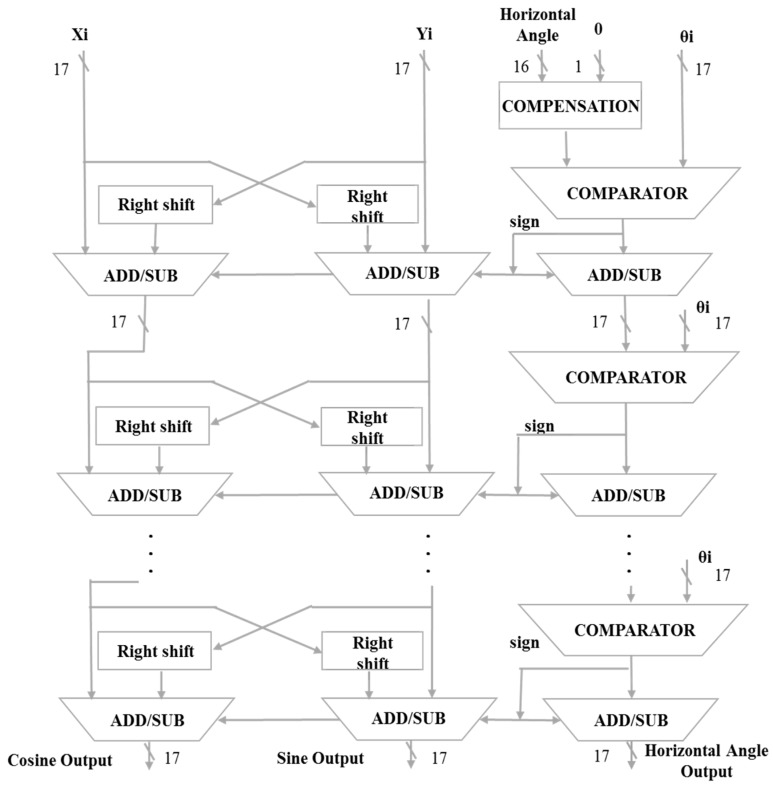
Horizontal angle iteration architecture.

**Figure 12:**
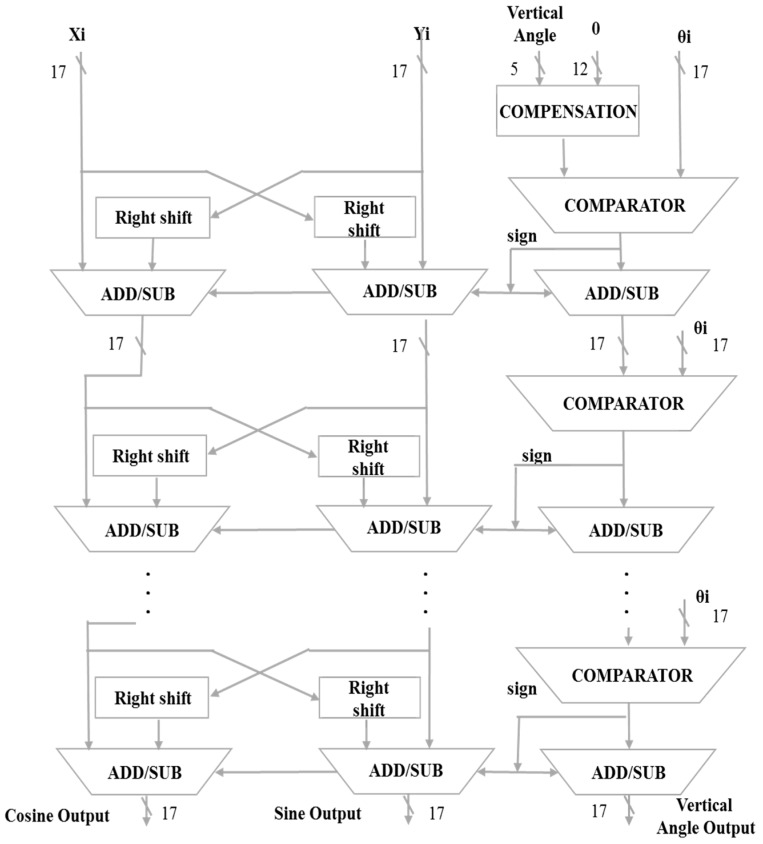
Vertical angle iteration architecture.

This architecture is a pipelined operation. When the operation proceeds from outputting values to the next iteration level, the data will be input at the same time. The number of layers in the iterative architecture is determined by selecting the angle of Table 3 and then determining the number of layers. However, since the hardware architecture cannot be different every time, the maximum number of rotations is fixed, at seven layers. ***Xi*** is the origin of the COS and substitutes 1. ***Yi*** is the origin of the SIN and is substituted for 0. ***Zi*** is the input angle, or the angle at which the SIN and COS values need to be found, and ***θi*** is the angle to be compared for input fixation. The highest bit of the input angle of ***Zi*** and the highest bit of the fixed input angle of ***θi*** are used as the ratio. This will determine the initial comparison input angle, After the comparison, we select the number of displacements ***i***, and determine the operation of the addition and subtraction of ***Xi*** and ***Yi***. After the calculation, the values ***Xi***, ***Yi*** and ***Zi*** continue to be substituted into the next-order iterative generation to repeat the operation until the value of the input angle ***Zi*** is less than 0.001, and the iterative operation is ended. When ***Xi***, ***Yi*** and ***Zi*** are converted into the second-order iterative generation from the first-order iterative generation, the data will be re-substituted into the first-order iterative generation to achieve timely operation, as shown in Figure 11 and Figure 12.

The CORDIC hardware circuit data processing is presented in Table 5 and Table 6 When the vertical angle or horizontal angle is input into the system, ***Xi*** and ***Yi*** will input the initial rotation value at the same time, and the data will be input continuously.

#### 4.3.5. Corrected Bit-Length Factor

**Figure 13:**
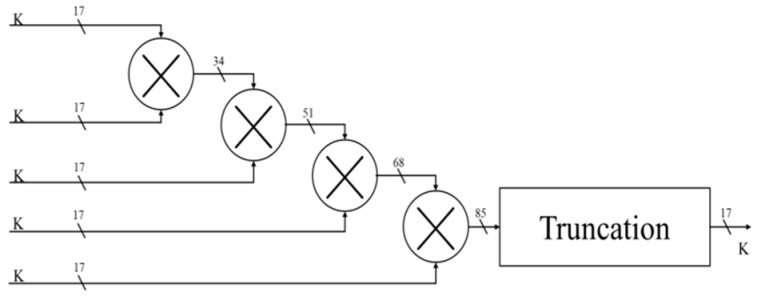
The total product of the K values.

**Figure 14:**
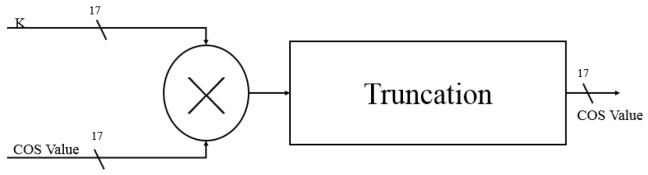
K value is multiplied by COS (cosine).

**Figure 15:**
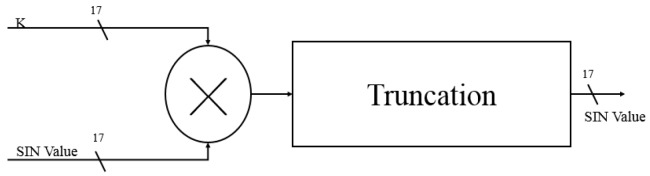
K value is multiplied by SIN (sine).

Since the angle of rotation is not fixed, the corrected bit-length factor will be different for each angle selection. According to the selection angle, the ***i*** is substituted into the formula, and the ***K*** value to be corrected for the current angle rotation is calculated (Figure 13). Finally, we multiply all the variables ***K*** and multiply the last rotated SIN and COS values to complete all angular rotations and obtain the correct SIN and COS values (Figure 14 and Figure 15). Each level multiplier uses a cycle so, in total, five cycles are used to calculate the total product of the ***K*** values.

**Figure 16:**
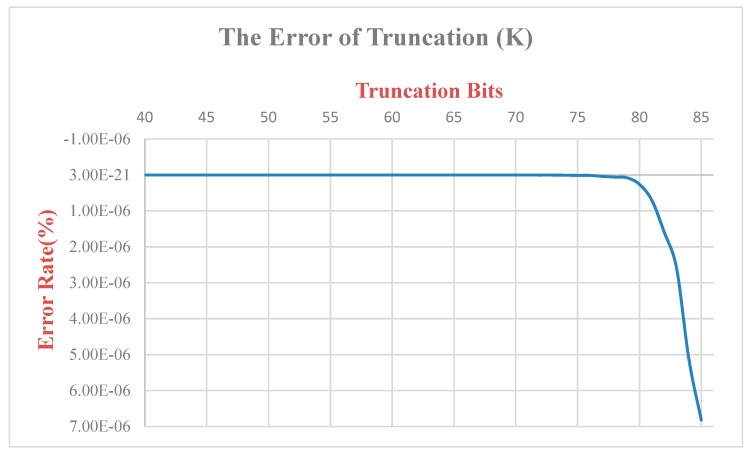
Truncated bit error rate-*K* value.

**Figure 17:**
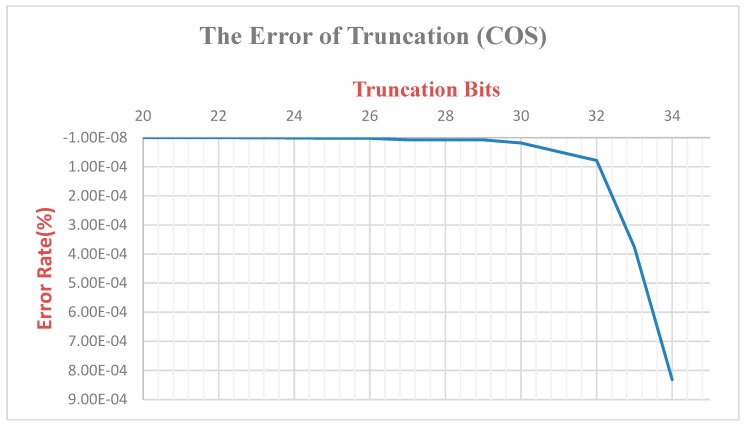
Truncated bit error rate-*COS* value.

**Figure 18:**
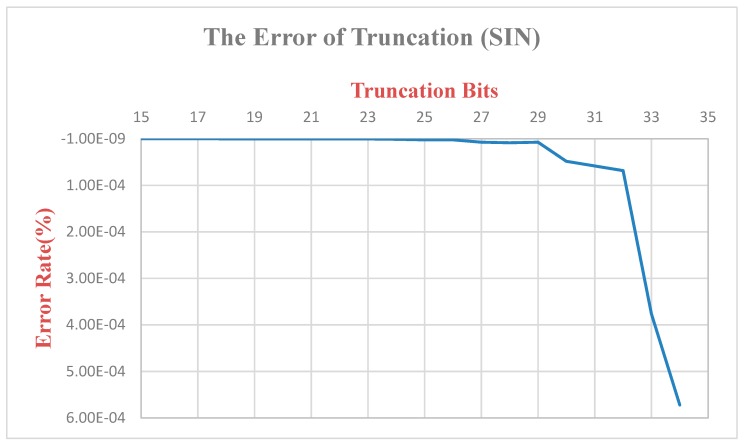
Truncated bit error rate-*SIN* value.

Since the ***K*** value is multiplied and there is truncation of the bit, the original output value will be different from the original one (Figure 16). Therefore, we calculate and analyze this difference. The K value has a total of 68 bits of truncation, the final error rate is 6.82 × 10^−6^, and the final output SIN and COS values are multiplied by the *K* value, respectively, so the truncation of the bits is 17 bits. The error rate of SIN is 5.72 × 10^−4^, and the error rate of COS is 8.32 × 10^−4^. Since the XYZ coordinate values output in this paper are only taken to the last three decimal places, they do not affect the post-coordinate values (Figure 17 and Figure 18).

#### 4.3.6. Coordinate Calculator

After the distance transmitted by the main controller and the values of SIN and COS calculated by the angle calculator are received, the information of the *X*-axis, the *Y*-axis, and the *Z*-axis is outputted in the coordinate calculator. Since the input timings of the horizontal angle, vertical angle, and distance are different, the final output timings of the *X*-axis, *Y*-axis, and *Z*-axis can be made uniform by the delay in the coordinate conversion calculator.

**Figure 19:**
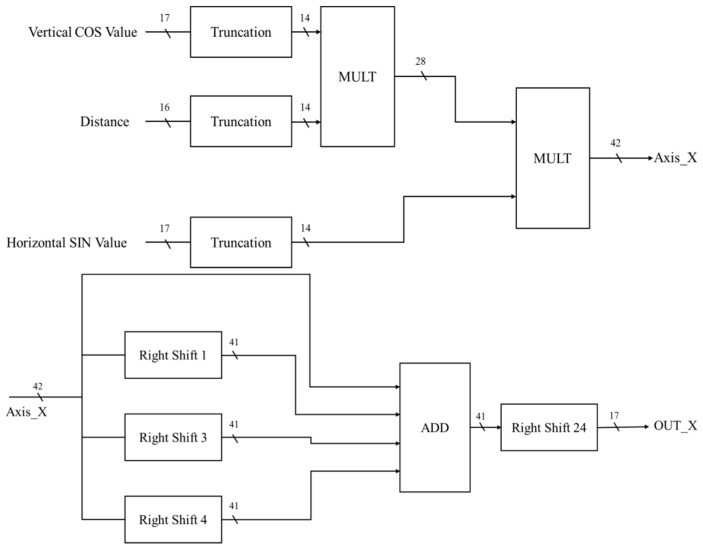
The *X*-axis circuit of the coordinate conversion calculator.

In the *X*-axis circuit of the coordinate conversion calculator, the COS value of the vertical angle, the SIN value of the horizontal angle, and the value of the distance are used as inputs (Figure 19). After truncation, the number of bits is 14 bits. The COS value of the vertical angle is multiplied by the value of the distance and then by the COS value of the vertical angle to obtain 42 bits. Since only the integer part of the three digits after the decimal is taken, it is necessary to divide the X axis by 10^7^. However, the because divider area is large, the displacement method can be used to achieve an approximate result, as shown in Equation (14).

(14) $${(2}^{0}{\;+\;2}^{-1}{+\;2}^{-3}{\;+\;2}^{-4}{)\;\times \;2}^{-24}\;=\;{1.0058\;\times \;10}^{-7}$$

**Figure 20:**
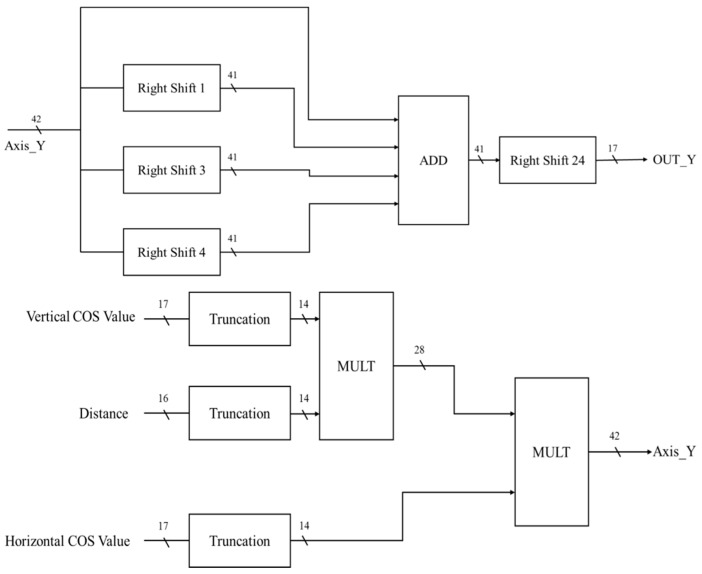
The *Y*-axis circuit of the coordinate conversion calculator.

In the *Y*-axis circuit of the coordinate conversion calculator, the input signal is the COS value of the vertical angle, the value of the COS value and the distance in the horizontal angle (Figure 20). After truncation, the number of bits is 14 bits. The COS value of the vertical angle is multiplied by the value of the distance and by the COS value of the vertical angle to obtain 42 bits. Since only the integer part of the three digits after the decimal is taken, it is necessary to divide the Y axis by $10^{7}$. To reduce the divider, we use a shift instruction to achieve an approximate result, as shown in Equation (15).

(15) $${(2}^{0}{\;+\;2}^{-1}{+\;2}^{-3}{\;+\;2}^{-4}{)\;\times \;2}^{-24}\;=\;{1.0058\;\times \;10}^{-7}$$

**Figure 21:**
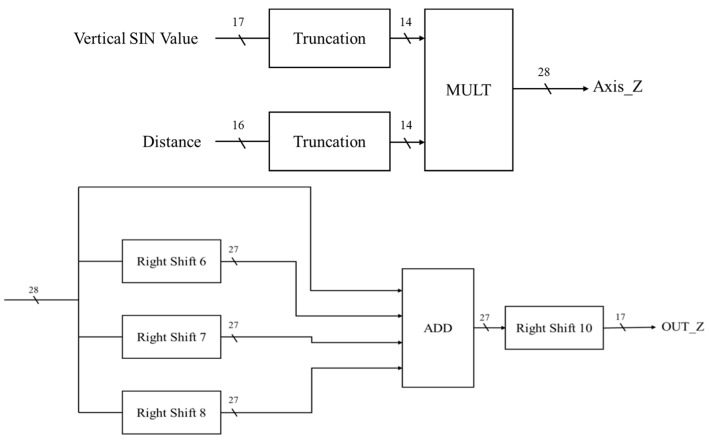
The *Z*-axis circuit of the coordinate conversion calculator.

In the *Z*-axis circuit of the coordinate conversion calculator, the SIN value of the vertical angle and the value of the distance are used as inputs (Figure 21). After truncation, the number of bits is 14 bits. The COS value of the vertical angle is multiplied by the value of the distance to obtain 28 bits, and the Z axis is divided by 10^3^. Approximate results can be achieved using the adder and shifting, as shown in Equation (16).

(16) $${(2}^{0}{+\;2}^{-6}{+\;2\;}^{-7}{\;+\;2}^{-8}{)\;\times \;2}^{-10}\;=\;{1.0032\;\times \;10}^{-3}$$

In this section, we introduce the LiDAR decoding circuit to detail the role of each module and implement the digital hardware architecture. Here we introduce the data transmission and operation process of each module, including the horizontal angle and vertical angle calculation. For the calculation of the distance, the trigonometric function reduces the iterative conversion method to the final coordinate, uses the reduced iteration method in the operation, and allows the data to be input into the new data at the same time during processing to achieve immediate processing.

## 5. Experimental Results

To prove the performance of the LiDAR sensing circuit designed in this paper, we have completed a series of experiments. We used LiDAR to sense different scenes and further decode the measured packet data. Then we performed point-cloud map conversion to verify the decoding results.

### 5.1. Experimental Environment

The hardware used in this experiment was a 16-channel LiDAR sensor, as shown in Table 7. The LiDAR sensor has a horizontal scanning angle of 360 degrees and a vertical scanning angle of +15 degrees to −15 degrees. The LiDAR sensor emits 16 infrared rays at a horizontal angle. According to the speed of the LiDAR sensor, the resolution of the horizontal angle is determined. The speed of the light is adjustable from 5 Hz to 20 Hz, and the corresponding horizontal angle is 0.1 to 0.4 degrees. Our experiment used the initial setting to simulate a rotational speed of 10 Hz and a horizontal resolution of 0.2 degrees. The LiDAR sensor can measure distances up to 100 m and sweep out 300,000 points per second.

### 5.2. Point-Cloud Map Reconstruction Experimental Results

We used the LiDAR sensor described in Section 5.1 and the LiDAR decoding algorithm described in Section 3 to convert the point-cloud image. We use the decoded 3D coordinate information as the reconstruction of the image and selected multiple scenes to present the decoding results.

**Figure 22:**
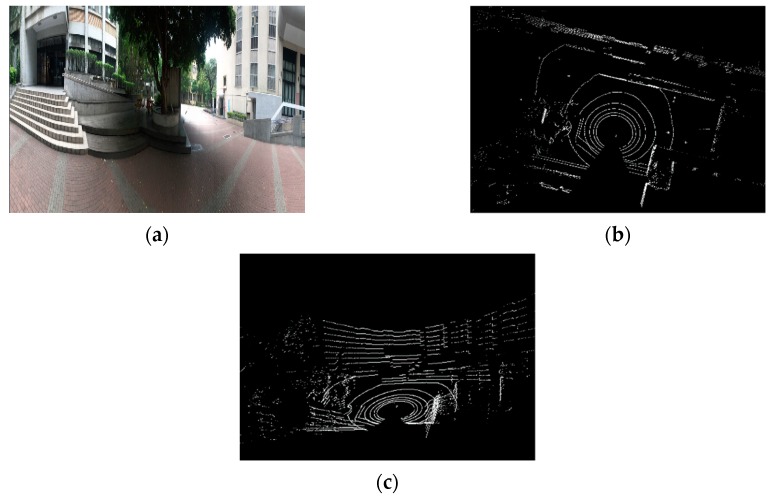
Point-cloud map reconstruction results: Square view. (**a**) Square view; (**b**) the point-cloud map of Square top view; (**c**) the point-cloud map of Square side view.

**Figure 23:**
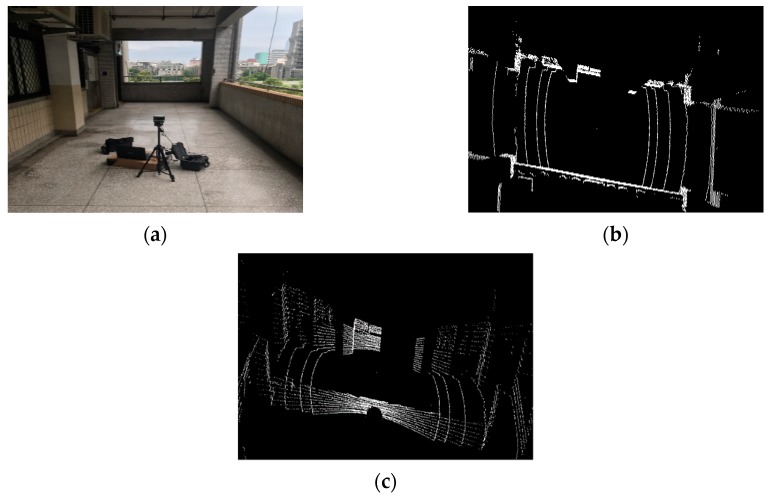
Point-cloud map reconstruction results: Corridor view. (**a**) Corridor view; (**b**) the point-cloud map of Corridor top view; (**c**) the point-cloud map of Corridor side view.

Figure 22a is a view of a square in our university. After decoding and reconstruction, Figure 22b shows the point-cloud map of the top view of the square, and Figure 22c shows the point-cloud map of the side view of the square. The side walls of buildings and large trees can be accurately measured by the LiDAR sensor and are clearly presented. Figure 23a shows a corridor view, and the clear outline of the corridor can be seen from the reconstructed point-cloud maps in Figure 23b,c.

We decoded the information in the LiDAR packet and reconstructed the 3D point-cloud image using different scenes. It can be observed in this section that the three-dimensional coordinates can be reconstructed smoothly indoors or outdoors, and point-cloud image reconstruction can be performed.

### 5.3. Processing Time

**Figure 24:**
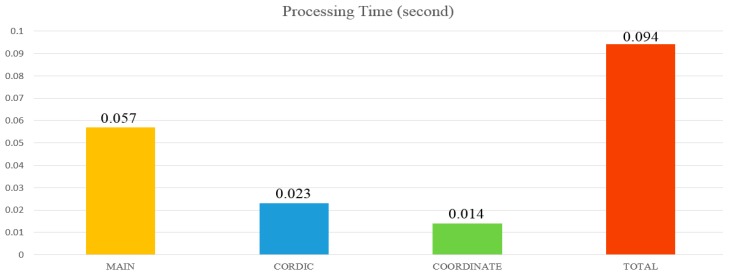
System operation time.

The LiDAR decoding process can be divided into three parts. The first part is the classification of the input data. The second part is the CORDIC algorithm, which mainly looks for the values of SIN and COS. The third part is the calculation of the XYZ coordinates. The processing times of these three blocks are different, and the total processing time is 0.094 s (Figure 24). To increase the overall circuit processing speed, the decoding circuit was designed as a decoding chip to improve the system processing time.

### 5.4. Integrated Circuit Implementation

In this section, we use the digital integrated circuit design flow to implement the circuit. The design flow includes register transfer level (RTL) hardware design, logic synthesis, testability circuit design, automated placement and routing, design rule check (DRC), layout versus schematic (LVS) and post-layout simulation. Then we use the TSMC 0.18 μm 1P6M process and implement the chip.

**Figure 25:**
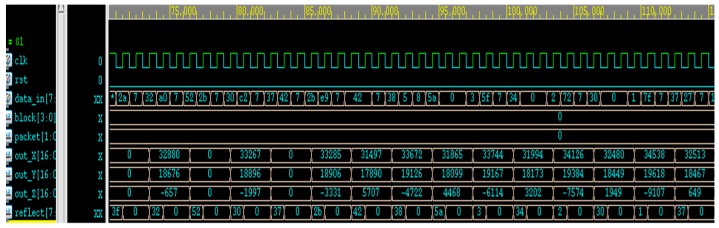
Register transfer level (RTL) timing diagram.

The value of the input signal can be observed from the waveform and the value of the output waveform is in accordance with the design expectation. In Figure 25, we can see the input data, the three-dimensional coordinates (out_X, out_Y, and out_Z), and reflectance values decoded by the chip. After calculation of the values of XYZ through the algorithm, the Golden Pattern is produced and the result is verified by Testbench.

**Figure 26:**
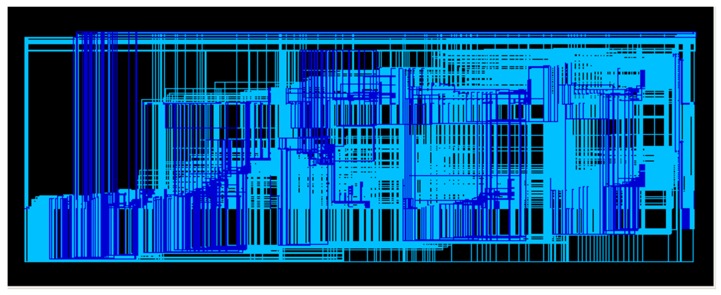
Gate-level circuit.

After the RTL code is verified, the next step is logic synthesis, which will convert the RTL code into a logic gate using the design compiler, as shown in Figure 26. In the logic synthesis, it is necessary to adjust the relevant environment settings, such as the timing, speed and related processes. Since the digital signal is not ideal, the problems of setup time and hold time need to be considered when the signal is changed.

**Figure 27:**
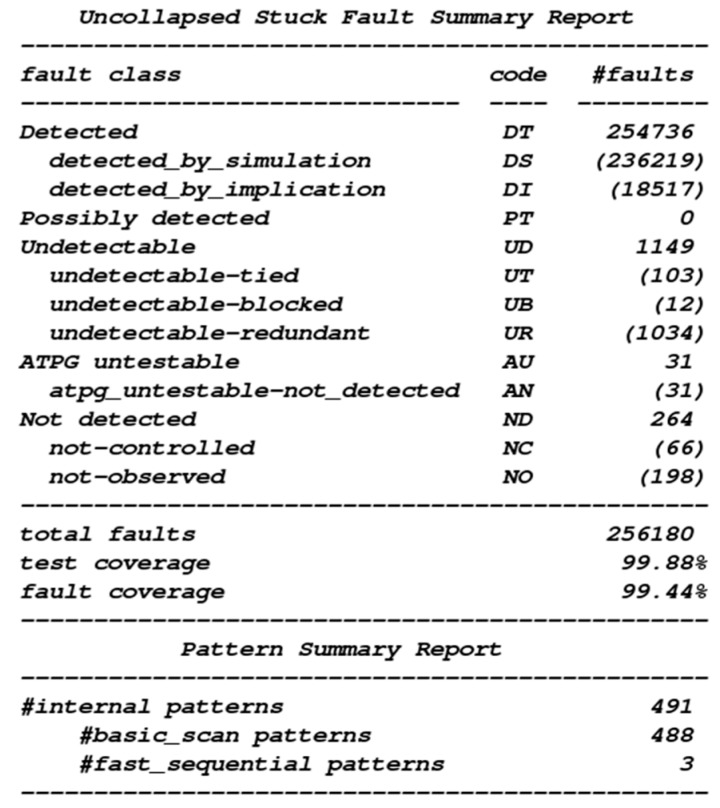
Test coverage and the fault coverage of the circuit.

The next step is the design of the testable circuit, mainly to facilitate the test of the main function of the chip after the chip is packaged. We verify the test coverage and the fault coverage of the circuit. The test coverage rate of this circuit is 99.88%, and the fault coverage rate is 99.44% (Figure 27).

**Figure 28:**
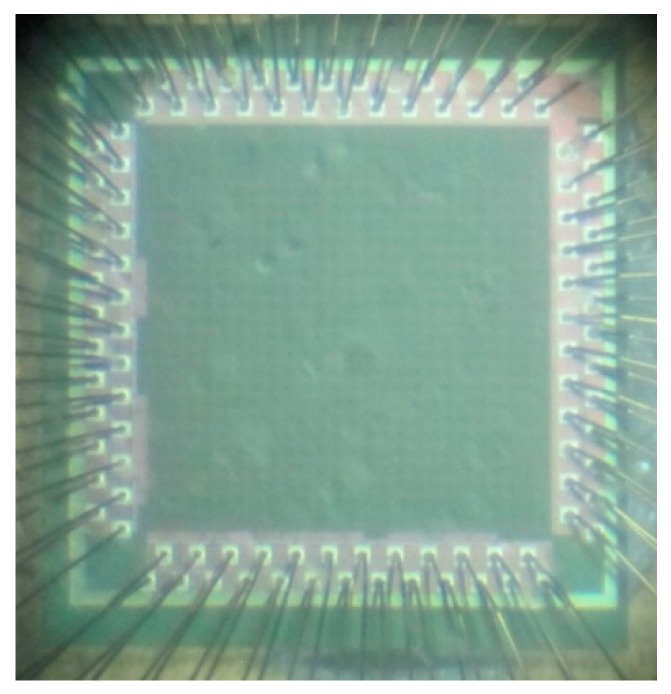
Microphotograph of the chip.

After backend design of the automatic placement and routing, DRC, LVS, and post-layout simulation, we adopt the TSMC 0.18 um 1P6M process to develop the chip. After manufacturing, the chip is packaged by CQFP 100 package. The total chip area is 1.93 mm × 1.93 mm, the core area is 1.32 mm × 1.32 mm, and the number of logic gates is 129,688. The maximum operating frequency is 100 MHz, and the average power consumption is 237.34 mW. The chip specifications are shown in Table 8, and a microphotograph of the chip is shown in Figure 28.

### 5.5. Chip Performance Comparison

This paper compares the performance of various CORDIC hardware architectures (Table 9). The first comparison is a look-up architecture circuit. Min [30] mainly uses the combined multi-constant multiplier (MMCM) and Wallace multiplier to perform the architecture. Both MMCM and fused butterfly with Wallace multiplier (FBW) adopt 45 nm VLSI processes. The operating frequency is 200 MHz, the areas are 101,312 μm^2^ and 116,886 μm^2^, and the power consumption of the chip is 127.13mW and 154.78 mW. Our experiment uses an 0.18 um VLSI process. According to the scaling factor rule of the VLSI process, if the chip is fabricated using a 45 nm process (Table 10), the operating frequency can be 400 MHz and the area is 54,997.6 μm^2^, and the power consumption is 2.656 mW.

The second comparison is with the standard CORDIC algorithm. Qi [25] uses the conversion between polar coordinates and rectangular coordinates to calculate the sine and cosine, which are rotated according to the standard method. Therefore, the maximum selection angle is 16 times, the process is 65 nm, the operating frequency is 35 MHz, the area is 863,300 μm^2^, and the power consumption is 145.33 mW. According to the scaling factor rule of the VLSI process, if the chip is fabricated using a 65 nm process, the operating frequency can be 276 MHz, the area is 109,995.25 μm^2^, and the power consumption is 5.312 mW.

Wu [26] proposed a CORDIC-based architecture that uses coordinate computer algorithms to achieve various operations. The process is 0.18 um, the maximum rotation is 16 times, and the operating frequency is 40 MHz. The area is 1,194,648 μm^2^ and the power consumption is 51 mW. Ray [27] mainly uses discrete Fourier transform to improve the calculation speed of CORDIC. The maximum operating frequency is 125 MHz, the total area is 11,000,000 μm^2^, the power consumption is 350 mW, and the number of rotations is 16.

The proposed chip is used to reduce the number of rotation iterations of the CORDIC calculation. After the angle input, the number of iterations can be reduced by more than half, and the area is also the smallest under the same process. Table 10 illustrates the process conversion table according to the VLSI scaling factor rule.

In this section, the proposed chip improves the traditional CORDIC calculator. Originally, it takes 0.094 s to calculate a packet and 7.05 s to decode a whole picture that has a total of 75 packets. The proposed chip takes only 0.012 ms to solve a packet and 0.912 ms to decode a frame. The throughput of the chip is 8.2105 ${\times \;10}^{8}$ bits/sec. The throughputs of the literature Qi [25], Wu [26] and Ray [27] are 1.2572 ${\times \;10}^{8}$ bits/sec, 1.4368 ${\times \;10}^{8}$ bits/sec and 4.4901 ${\times \;10}^{8}$ bits/sec respectively. The method we have proposed to reduce the number of iterations, the performance of throughput is much higher than other methods in the literature [25,26,27].

To verify that the reconstructed point clouds are correct, we compare with Velodyne LiDAR VLP-16 ground truth data that includes County Fair, Hecker Pass, and Monterey Highway. Moreover, in order to compare the correctness of the decoding of LiDAR packet data, we compare the decoding results of the standard test environment of VeloView 2.0. In April 2014, Velodyne and Kitware collaborated to release VeloView 2.0 in SPAR International 3D Measurement and Imaging Conference. VeloView displays the distance measurements from the LidAR as point-cloud data such as intensity-of-return, time, distance, azimuth, dual return type, and laser ID. Correctness verification is performed using a correlation criterion in which the “similarity” between the LiDAR decoded packet data s and the ground truth w, Sim(s, w), is calculated as follows:

(17) $$Sim(s,w)=\frac{\sum_{i=1}^{m}\left(s_{i}\cdot w_{i}\right)}{\sum_{i=1}^{m}\left(w_{i}\cdot w_{i}\right)}$$

We calculate the similarity value. The results are included in Table 11. The experimental results show the Sim values all to be 1. The experimental results prove that the LiDAR decoded packet results are exactly the same as the ground truth.

## 6. Conclusions

An efficient CORDIC iteration design for LiDAR sensors and point-cloud map reconstruction technology are proposed in this paper. A new CORDIC architecture is designed for LiDAR sensors to improve the traditional CORDIC architecture by changing the rotation characteristics to achieve the goal of pre-selecting angles and reducing the number of iterations. With the proposed architecture, we can reduce the number of iterations by half. Regardless of the number of input N digits, the values of the trigonometric functions SIN and COS can be found in seven rotations. According to the SIN and COS values obtained and distance information, the 3D point-cloud image is obtained through the three-dimensional coordinate conversion system.

To prove the performance of the presented LiDAR sensing circuit, we have completed a series of experiments. The proposed chip improves the traditional CORDIC calculator. The proposed chip takes only 0.012 ms to solve a packet and 0.912 ms to decode a frame. The fault coverage rate of the proposed chip is 99.44%. The total chip area is 1.93 mm × 1.93 mm and the core area is 1.32 mm × 1.32 mm. The number of logic gates is 129,688. The maximum operating frequency is 100 MHz, and the average power consumption is 237.34 mW. Compared with those in related literature, our proposed sensing circuit has smaller area and low power consumption.

## Figures and Tables

**Table 1:** Mobile Light Detection and Ranging (LiDAR) packet rate.

Mode	Packets/Sec	Mbits/Sec
Strongest Return Mode	754	8
Last Return Mode	754	8
Dual Return Mode	1508	16

**Table 2:** LiDAR vertical angle.

Laser ID (Channel)	Vertical Angle	Laser ID (Channel)	Vertical Angle
0	−15°	8	−7°
1	+1°	9	+9°
2	−13°	10	−5°
3	+3°	11	+11°
4	−11°	12	−3°
5	+5°	13	+13°
6	−9°	14	−1°
7	+7°	15	+15°

**Table 3:** The rotation angle of the traditional CORDIC algorithm.

i	θ	i	θ
0	45.000000000	8	0.223810500
1	26.565051177	9	0.111905677
2	14.036243468	10	0.055952892
3	7.125016349	11	0.027976453
4	3.576334375	12	0.013988227
5	1.789910608	13	0.006994114
6	0.895173710	14	0.003497057
7	0.447614171	15	0.001748528

**Table 4:** Correction factor for different iterations.

i	K	i	K
0	0.707106781	8	0.999992371
1	0.894427191	9	0.999998093
2	0.970142500	10	0.999999523
3	0.992277877	11	0.999999881
4	0.998052578	12	0.999999970
5	0.999512076	13	0.999999993
6	0.999877952	14	0.999999998
7	0.999969484	15	0.999999999

**Table 5:** CORDIC horizontal angle architecture data flow.

Clock Cycle	Data Sequences											
	X_i(i=1)_	X_i(i=2)_	X_i(i=3)_	X_i(i=4)_	X_i(i=5)_	X_i(i=6)_	Y_i(i=1)_	Y_i(i=2)_	Y_i(i=3)_	Y_i(i=4)_	Y_i(i=5)_	Y_i(i=6)_
0	inX_1_						inY_1_					
1	inX_2_	inX_1_					inY_2_	inY_1_				
2	inX_3_	inX_2_	inX_1_				inY_3_	inY_2_	inY_1_			
3	inX_4_	inX_3_	inX_2_	inX_1_			inY_4_	inY_3_	inY_2_	inY_1_		
4	inX_5_	inX_4_	inX_3_	inX_2_	inX_1_		inY_5_	inY_4_	inY_3_	inY_2_	inY_1_	
5	inX_6_	inX_5_	inX_4_	inX_3_	inX_2_	inX_1_	inY_6_	inY_5_	inY_4_	inY_3_	inY_2_	inY_1_
. . .	. . .	. . .	. . .	. . .	. . .	. . .	. . .	. . .	. . .	. . .	. . .	. . .
2399995	inX_t-1_	inX_t-2_	inX_t-3_	inX_t-4_	inX_t-5_	inX_t-6_	inY_t-1_	inY_t-2_	inY_t-3_	inY_t-4_	inY_t-5_	inY_t-6_
2399996		inX_t-1_	inX_t-2_	inX_t-3_	inX_t-4_	inX_t-5_		inY_t-1_	inY_t-2_	inY_t-3_	inY_t-4_	inY_t-5_
2399997			inX_t-1_	inX_t-2_	inX_t-3_	inX_t-4_			inY_t-1_	inY_t-2_	inY_t-3_	inY_t-4_
2399998				inX_t-1_	inX_t-2_	inX_t-3_				inY_t-1_	inY_t-2_	inY_t-3_
2399999					inX_t-1_	inX_t-2_					inY_t-1_	inY_t-2_
2400000						inX_t-1_						inY_t-1_

**Table 6:** CORDIC vertical angle architecture data flow.

Clock Cycle	Data Sequences											
	X_i(i=1)_	X_i(i=2)_	X_i(i=3)_	X_i(i=4)_	X_i(i=5)_	X_i(i=6)_	Y_i(i=1)_	Y_i(i=2)_	Y_i(i=3)_	Y_i(i=4)_	Y_i(i=5)_	Y_i(i=6)_
0	inX_1_						inY_1_					
1	inX_2_	inX_1_					inY_2_	inY_1_				
2	inX_3_	inX_2_	inX_1_				inY_3_	inY_2_	inY_1_			
3	inX_4_	inX_3_	inX_2_	inX_1_			inY_4_	inY_3_	inY_2_	inY_1_		
4	inX_5_	inX_4_	inX_3_	inX_2_	inX_1_		inY_5_	inY_4_	inY_3_	inY_2_	inY_1_	
5	inX_6_	inX_5_	inX_4_	inX_3_	inX_2_	inX_1_	inY_6_	inY_5_	inY_4_	inY_3_	inY_2_	inY_1_
. . .	. . .	. . .	. . .	. . .	. . .	. . .	. . .	. . .	. . .	. . .	. . .	. . .
2399995	inX_t-1_	inX_t-2_	inX_t-3_	inX_t-4_	inX_t-5_	inX_t-6_	inY_t-1_	inY_t-2_	inY_t-3_	inY_t-4_	inY_t-5_	inY_t-6_
2399996		inX_t-1_	inX_t-2_	inX_t-3_	inX_t-4_	inX_t-5_		inY_t-1_	inY_t-2_	inY_t-3_	inY_t-4_	inY_t-5_
2399997			inX_t-1_	inX_t-2_	inX_t-3_	inX_t-4_			inY_t-1_	inY_t-2_	inY_t-3_	inY_t-4_
2399998				inX_t-1_	inX_t-2_	inX_t-3_				inY_t-1_	inY_t-2_	inY_t-3_
2399999					inX_t-1_	inX_t-2_					inY_t-1_	inY_t-2_
2400000						inX_t-1_						inY_t-1_

**Table 7:** LiDAR sensor specification.

Specification	Value
Channel	16
Measurement Range	100 m
Accuracy	±3 cm
Laser	903 nm Wavelength
Horizontal Angle	360°
Vertical Angle	30° (+15° to −15°)
Rotation Rates	5–20 Hz
Power Consumption	8 Watt
Operating Temperature	−10° to +60°

**Table 8:** Chip specification.

Specification	Value
Technology	TSMC 0.18 μm 1P6M
Chip Size	1.93 mm × 1.93 mm
Core Size	1.32 mm × 1.32 mm
Package	CQFP 100
Gate Counts	129,688
Frequency	100 MHz
Scan Chain	1
Fault Coverage	99.44%
Power Supply	1.8V
Power Consumption	237.34 mW@100 MHz

**Table 9:** Comparison of hardware performance between CORDIC circuit and related literature.

Method	Process	Type	Architecture	Frequency (MHz)	Max Iteration	Throughput Bits/Sec
MMCM [30]	45 nm	Chip	LUT	200	-	-
FBW [30]	45 nm	Chip	LUT	200	-	-
Qi [25]	65 nm	Chip	CORDIC	35	16	1.2572 ${\times \;10}^{8}$
Wu [26]	0.18 um	FPGA	CORDIC	40	16	1.4368 ${\times \;10}^{8}$
Ray [27]	0.18 um	Chip	CORDIC	125	16	4.4901 ${\times \;10}^{8}$
Proposed	0.18 um	Chip	CORDIC	100	7	8.2105 ${\times \;10}^{8}$

**Table 10:** Chip scaling.

Process	Frequency	Chip Area	Power
90 nm	200 MHz	219,990.00 μm^2^	10.625 mW
65 nm	276 MHz	109,995.25 μm^2^	5.312 mW
45 nm	400 MHz	54,997.60 μm^2^	2.656 mW

**Table 11:** The correctness of the decoding of LiDAR packet data.

Ground Truth	Header	Flag	Azimuth	Distance	Reflectivity	Time Stamp	Factory	Return Distance
County Fair	1	1	1	1	1	1	1	1
Hecker Pass	1	1	1	1	1	1	1	1
Monterey Highway	1	1	1	1	1	1	1	1

Similarity: Sim(s, w).
